# Burnout, Reasons for Living and Dehumanisation among Italian Penitentiary Police Officers

**DOI:** 10.3390/ijerph17093117

**Published:** 2020-04-30

**Authors:** Ines Testoni, Irene Nencioni, Lucia Ronconi, Francesca Alemanno, Adriano Zamperini

**Affiliations:** 1 FISPPA Department, University of Padova, 35139 Padova, Italy; irene.nencioni4@gmail.com (I.N.); l.ronconi@unipd.it (L.R.); adriano.zamperini@unipd.it (A.Z.); 2 Emili Sagol Creative Arts Therapies Research Center, University of Haifa, Haifa 349883, Israel; 3 European and Mediterranean Cultures (DiCEM) Department, University of Basilicata, 75100 Matera, Italy; francesca.alemanno@unibas.it

**Keywords:** prison, penitentiary police officer (PPO), burnout syndrome, reasons for living, de-humanisation, workplace well-being

## Abstract

The literature on burnout syndrome among Penitentiary Police Officers (PPOs) is still rather scarce, and there are no analyses on the protective factors that can prevent these workers from the dangerous effect of burnout, with respect to the weakening of the reasons for living and de-humanization. This study aimed to examine the relationships between burnout, protective factors against weakening of the reasons for living and not desiring to die and the role of de-humanisation, utilising the Maslach Burnout Inventory (MBI); the Reasons for Living Inventory (RFL); the Testoni Death Representation Scale (TDRS); and the Human Traits Attribution Scale (HTAS), involving 86 PPOs in a North Italy prison. Results showed the presence of a high level of burnout in the group of participants. In addition, dehumanization of prisoners, which is considered a factor that could help in managing other health professional stress situations, does not reduce the level of burnout.

## 1. Introduction

During the last decades in the Western countries, growing attention has been paid to workplace well-being, which is a construct related to all aspects of working life, from the quality and safety of the physical environment to the climate at work and how workers feel about their activities, to improve the organisation and ensure that workers are safe, healthy, satisfied and engaged at work. This attention arose from discoveries about the phenomenon of the burnout syndrome, which is a work-related set of symptoms that arises in response to chronic interpersonal and emotional job stressors [1]. The term describes a state of exhaustion resulting from permanent contact with suffering or needy people [2]. It usually occurs in individuals without any prior history of psychological or psychiatric disorders, especially in those who carry out so-called helping professions, the aim of which is to take care of people [1]. It starts with a feeling of emotional stress and decreasing job satisfaction that escalates into negative attitudes towards the job, colleagues and clients/users [3]. Christina Maslach described this discomfort as a specific syndrome characterised by three components [4]: ‘emotional exhaustion’ due to prolonged and systematic contact with the users. It is a feeling of constant tension and lack of mental and physical energy at work, and an inability to regain strength and face new projects. ‘Cynicism and detachment’, involving uncaring feelings, emotional detachment and a cynical attitude towards the job and people. Finally, ‘reduced personal accomplishment’, consisting of the loss of feeling competent at work, meaning the operator feels incapable and inadequate at work. The syndrome has received great attention with respect to its effects on health and well-being [5,6]. Research has widely showed how burnout causes a multiplicity of problems at work such as job dissatisfaction, absenteeism, dismissals and abandonment of jobs [7,8], with negative physical, psychological and social effects on the personal well-being of workers, which include suicidal ideation and attempts [6,9]. Although to date the direct relationship between burnout and suicide has not been extensively investigated, some studies have shown that health services professionals such as physicians, dentists and nurses have higher-than-average suicide rates compared to other occupations and the general population [10,11]. In addition, individuals with relatively high frequencies of burnout met the diagnostic criteria for depression [12,13]. Recent studies have found, on the one hand, a high rate of suicidal ideation in teachers with burnout who met the criteria for a provisional diagnosis of depression [14], and on the other hand, that burnout appears to be an important mediator in understanding veterinarians’ suicidal tendencies, too [10]. 

More recently, another significant field of research has been developing in medical and health contexts that pay particular attention to the relationships between burnout and dehumanisation, which has become an expanding area of research in social psychology [15]. Dehumanisation consists in viewing and treating other persons as if they lack the characteristics that we enjoy as human beings, such as cognitive abilities and reasoning; in doing this, people are able to manage stress situations, such as the death of a patient in medical field [15]. Some scholars have considered the reasons why health professionals may offend the patients’ dignity or enact forms of more or less aggressive behaviour towards them. Vaes and Muratore’s [16] study about healthcare professionals showed that dehumanisation was positively related to higher work engagement and perceived professional efficacy. This result shows that dehumanisation may be related to unconscious attitudes towards the patients by health professionals with a self-defensive role, as a protective factor against burnout. In fact, the consequence is that they are able to provide better care to patients [17]. Trifiletti et al. [18] reported similar results, finding that humanisation of attitudes towards patients was related to higher levels of stress, while dehumanisation was related to lower levels of stress and furthermore to emotional involvement, commitment at work, and efficiency. 

From the 1980s, research on burnout has been applied to different organisational environments, and many other professional categories have been considered potentially stressful and at risk (e.g., managers, engineers, lawyers, white-collar professions, and policemen). The studies on police officers run in parallel with the other kinds of workers [19,20,21,22,23], and the literature has already confirmed that this category is one of the most at risk of developing burnout, with very important negative consequences to the personal well-being of policemen such as divorce, alcohol addiction, physical and psychological health problems [24,25], post-traumatic stress symptoms, cognitive effects and suicidal ideation [26]. Griffin et al. [27], using the Maslach Burnout Inventory [4], showed that younger police officers were characterised by using ‘depersonalisation’ more than older ones, which is an important dimension of burnout. Finney et al.’s [28] meta-analysis of the organisational stressors related to high levels of stress and burnout in Penitentiary Police Officers (PPOs) highlighted several crucial factors: job stressors (e.g., work overload, tasks, training), role ambiguity, rewards at work (internal such as personal satisfaction and external such as recognition for the work done), bad quality of communication between supervisors and operators and a negative organisational climate. 

In 1997, Stack and Tsoudis [29] conducted the first study of suicide risk among PPOs. Their analysis of data from 21 US states indicated that the risk of suicide among prison officers is 39% higher than that of the rest of the working population. The authors hypothesised that high levels of job dissatisfaction and stress might be the causes. Unfortunately, there are no official data or specific studies about numbers and motives of PPOs suicides. What is certain is that some studies have confirmed that the problem of burnout among PPOs is particularly significant in Italy as well [26,30]. From 2010 to 2018, about 59 prison officers committed suicide [31]. In the Italian scenario, the reason for the high level of PPOs’ stress may be related to the changes in the prison conditions, which have increased the level of complexity and strain on their role. Many difficulties have arisen related to the growing increase of prisoners’ numbers and the cultural diversification of the population due to immigration. Furthermore, in Italy, Law 395/1990 established that PPOs have to take part in inmates’ treatment and rehabilitation programmes, and since 2015, when the judicial psychiatric hospitals were closed, they have had to deal with psychological pathologies without any psychological competence. Some studies have shown that the most common perceptions of PPOs are that of a strong organisational inequity, lack of recognition at work and discomfort with societal judgement, whereas the fear of being seen as those who torture prisoners runs parallel with worry about being socially juxtaposed with prisoners [32,33] and is related to anger and frustration with the perception of their low social prestige [26]. Indeed, PPOs receive inadequate training to cope with job-related stress, both during and after highly emotional events, so the longer an officer is exposed to stress, the faster this exposure can lead to aggression, anxiety and poor impulse control. However, in Italy, studies are still quite scarce on suicide among these workers, and there are no studies of their possible dehumanisation attitudes towards prisoners. All this is particularly important, especially if considered in relationship with Gilmartin’s study [34], which showed that violence, horrific scenes and other emotional and traumatic events are considered routine and part of the job by men and women in law enforcement professions. This research reflects on these variables, considering PPOs’ work a specific area that requires important psychological interventions to support their well-being.

In Italy, the number of studies about work-related stress, burnout and dehumanisation in a penitentiary context is very small. Researches and information about this particular social field are rather scarce, especially because of the difficulty of getting in touch with the world of the prison. Especially regarding the burnout and suicide risk of Penitentiary Police Officers (PPOs), there is almost nothing. However, the problem is real, and the penitentiary administration know it so well.

This study dealt with the presence of burnout among Italian PPOs and its possible relationships with suicide risk and dehumanisation of prisoners. In particular, it focused on the importance assigned to reasons for avoiding suicide (possible scarcity of protective factors against suicide) and the possible presence of dehumanisation towards inmates as a protective factor. Following the literature on burnout, suicide and dehumanisation, the hypothesis was that the higher gravity of burnout was, the lower the protective factors were. Moreover, since there is no specific literature on dehumanisation and burnout among PPOs, we wanted to describe the role of dehumanisation towards inmates and colleagues. The further aim was to evaluate the impact on severity of burnout of several predictor variables by three different steps: first, considering only personal variables; second, examining the contribution of working life variables controlled for personal variables; third, analysing the role of the reasons for living and not committing suicide controlled for personal and working variables.

## 2. Materials and Methods 

The study involved 86 PPOs (73 men, 5 women and 8 not specified; mean age = 40.3) who have been working with prisoners in a North Italian prison for at least one year. Most of the participants (63 = 73%) came from the centre or south of Italy; 36 (42%) had a low level of education; 76 (88%) were Christians and the remaining 10 (12%) were atheists; 62 (72%) were married or lived with a partner; and 56 (65%) had at least one child. Table 1 reports participants’ personal and work characteristics.

In July 2018, one researcher introduced the objectives of the study, the further researchers involved and the research design and the informed consent to the Ethics committee, to the director of the prison and to the PPOs during an assembly.

After receiving all the permissions, the data were collected in the winter between 2018–2019, in a dedicated room (a welcoming meeting point for breaks) of the prison. All the requirements and demands of the Director of the prison were respected: in particular, the recruitment of voluntary participants, who could fill the questionnaire in their breaks, when the researcher was present to give them all the further necessary explanations and support (8:00 a.m. to 4:00 p.m.) After their approval, the informed consent form was distributed and participants completed the questionnaire, consigning all the material to the researcher. 

The Italian version of the Maslach Burnout Inventory (MBI) (Maslach & Jackson, 1981), realized by Sirigatti and Stefanile (1983), was used [35]. The instrument consists of 22 statements about personal attitudes and feelings, scored on a 7-point Likert scale ranging from (0) = ‘never’ to (6) = ‘every day’. The inventory assesses the three components of burnout according to the authors: emotional exhaustion (e.g., ‘I feel emotionally exhausted from my work’), depersonalisation and personal realisation. 

The Reasons for Living Inventory (RFL) [36] is a 48-item, self-report instrument designed to evaluate a range of adaptive beliefs, motivations and expectations for living if suicide is considered. The inventory has six subscales: Survival and Coping Beliefs (e.g., ‘I believe I can find a purpose in life, a reason to live’), Responsibility to Family (e.g., ‘My family depends on me and needs me’), Child-Related Concerns (e.g., ‘I want to watch my children as they grow’), Fear of Suicide (e.g., ‘I am afraid of the ‘act’ of killing myself (the pain, the blood and violence’), Fear of Social Disapproval (e.g., ‘I am concerned about what other people think of me’) and Moral Objections related to suicide (e.g., ‘I consider it morally wrong’). The 48 items are scored on a 6-point scale ranging from (1) = ‘not at all important’ to (6) = ‘extremely important’. Higher scores represent more reasons to live. The RFL also indicates which factors must be considered when planning suicide prevention programmes [37]. This questionnaire was chosen because it emphasises cognitive adaptation and positive traits as protective factors against suicidal ideation, instead of focusing on negative pressure that could bring to suicide acts [38]. 

The Testoni Death Representation Scale (TDRS) [39] investigates the different representations of death. The Scale compares a representation of death as an absolute annihilation (after the death, there is nothing and the beings become nothing), with a representation of death as a passage (after the death there is something else and who dies simply changes and reaches another existential dimension). The scale consists of six items scored on a 5-point Likert scale. The TDRS resulted to be correlated to several variables concerning psychological suffering: people who consider death a total annihilation of individual and personal identity (higher score) tend to lose hope in the future and to have lower resilience [39]. Conversely, according to the literature, a person who considers death a passage (lower score) will have a greater degree of resilience and, therefore, greater protection from suicidal thoughts. This scale was chosen to investigate whether an eventual presence of nihilism is related to burnout and suicide risk, as shown by a recent study [39].

The Human Traits Attribution Scale (HTAS) [40] investigates the attribution of human and non-human traits both to in-group members (PPOs) and to members of the out-group (prisoners). This instrument consists of eight items scored on a 5-point Likert scale ranging from (1) = ‘does not describe at all’ to (5) = ‘describes very much’. The eight items describe personality traits divided into two categories: four uniquely human traits (‘reasoning’, ‘thinking’, ‘cognitive abilities’ and ‘moral sense’) and four uniquely non-human traits (‘impetus’, ‘impulsiveness’, ‘instinct’ and ‘impulses’). In this study, two human traits attribution scales were used: one refers to the prisoners (e.g., ‘In your opinion, how much are the prisoners characterised by reasoning/impetus’) and the other to the staff of PPOs (e.g., ‘According to you, how much are your colleagues characterised by reasoning/impetus’).

Preliminary analysis to check reliability and distribution of study variables were conducted. Cronbach’s alpha was used as measure of reliability, or internal consistency, of each test items set. Values ranged from 0.71 to 0.92, which indicated an adequate reliability for all measures. Skewness and kurtosis statistics divided by their standard errors ranged from −2.5 to 2.5 which indicated no significant skew and a distribution of the scores close to the normal distribution. Only four variables exceed the range −2.5 to 2.5 for skewness (Survival and coping beliefs, Child-related concern, Fear of suicide and Fear of social disapproval) and only one variable exceed the range for kurtosis (Child-related concern). First, we examined the presence of burnout in our sample using the critical values for the total scores of each component of burnout reported in the Italian validation of MBI [35]. In particular, we considered problematic scores over 23 for emotional exhaustion, over 3 for depersonalization and less than 34 for personal realization. Next, we investigated which reasons for living and representation of death are more important as protective factors to stress and suicide. Then, we evaluated the difference in human/non-human traits attribution between in-group and out-group. Then, we observed correlations between all study variables and with personal and work variables. Finally, we analysed the impact of personal variables, work variables and RFL variables on MBI subscales, performing several regression analyses. Personal variables with a correlation of over 0.16 with at least one of three MBI subscales are included in the first regression model. Work variables with a correlation of over 0.16 with at least one of three MBI subscales are added to personal variables in the second regression model. RFL variables with a correlation of over 0.16 with at least one of three MBI subscales are also added to personal and work variables in the last regression model. To evaluate the fit of each regression model R-square, i.e., the quote of variance of dependent variable explained by the model, and F-test results for R-square were considered. Moreover, residual plots were examined to check residual distribution assumptions. For all models standardized residual scores ranged from −2.5 to 2.5 which indicated a residual distribution close to the normal distribution. The analyses were conducted on all participants to provide an overall view of the results with SPSS 25 (IBM Corp. Released 2017. IBM SPSS Statistics for Windows, Version 25.0. IBM Corp, Armonk, NY, USA). We also replicated all analyses for males only (see Supplementary Tables S1 and S2) to prove that the few female participants did not influence the fundamental results of the study.

## 3. Results

With respect to burnout, 30% of participants show high levels of emotional exhaustion, 60% show high levels of depersonalisation and 17% show lower levels of personal realisation. Overall, 25 participants, about 30% of the sample, showed the presence of burnout, with high levels of both emotional exhaustion and depersonalisation. With respect to the RFL: Child-Related Concern, Survival and Coping Beliefs and Responsibility to Family are the reasons for living with a higher score; in contrast, Fear of Suicide, Fear of Social Disapproval and Moral Objection are the reasons for living with a lower score. With regard to the representation of death, the average score is close to the central point of the scale, and therefore, PPOs do not seem oriented either towards a vision of death as a passage or towards the opposed vision of total annihilation. There is a significant difference in the attribution of human and non-human traits between in-group and out-group, with more human traits attributed to in-group (t = 9.27 df = 85 *p* < 0.001) and more non-human traits to out-group (t = 7.74 df = 85 *p* < 0.001). 

### 3.1. Correlations

Emotional exhaustion and depersonalisation are negatively correlated with survival and coping beliefs (r = −0.39 *p* < 0.001 and r = −0.21 *p* = 0.052, respectively); they are positively correlated with fear of suicide (r = 0.19 *p* = 0.076 and r = 0.36 *p* = 0.001, respectively). Moreover, depersonalisation is positively correlated with fear of social disapproval and responsibility to family (r = 0.26 *p* = 0.016 and r = 0.23 *p* = 0.033, respectively). Personal realisation is negatively correlated with fear of suicide (r = −0.20 *p* = 0.069) and is positively correlated with the human traits attributed to the in-group (r = 0.24 *p* = 0.025). Fear of suicide is negatively correlated with the human traits attributed to the in-group (r = −0.22 *p* = 0.040), and child-related concern is positively correlated with non-human traits attribution to out-group (r = 0.22 *p* = 0.038). Death being represented as total annihilation is negatively correlated with child-related concern and moral objection (r = −0.25 *p* = 0.022) (Table 2).

### 3.2. Regression Analyses

To analyse the impact of personal variables, work variables and RFL variables on MBI subscales, several regression analyses were performed. In the first model, only personal variables are included; in the second model, work variables are added to personal variables; and in the last model, RFL variables are added to personal and work variables (Table 3). The first regression model shows a non-significant portion of variance of MBI subscales (R-square = 0.05; R-square = 0.06 and R-square = 0.06, respectively), indicating a non-relevant role for personal variables in explaining burnout. The second regression model shows a significant portion of variance only for depersonalisation (R-square = 0.17), indicating a partially relevant role for work variables in burnout explanation. Finally, the third regression model shows a significant portion of variance for both emotional exhaustion and depersonalisation (R-square = 0.36 and R-square = 0.37, respectively), indicating a relevant role for RFL variables in explaining burnout. In the last model, we observe significant positive beta coefficients for length of service (β = 0.19 *p* = 0.067 on EE), working time (β = 0.17 *p* = 0.090 on DP), responsibility to family β = 0.34 *p* = 0.009 on EE and β = 0.22 *p* = 0.083 on DP) and fear of suicide (β = 0.24 *p* = 0.077 on DP); consequentially, they are risk factors for burnout. On the contrary, we observe significant negative beta coefficients for presence of religious practice (β = −0.20 *p* = 0.040 on DP) and survival and coping beliefs (β = −0.56 *p* < 0.001 on EE and β = −0.39 *p* = 0.001 on DP). Consequentially, they are protective factors for burnout.

## 4. Discussion

The results showed that some participants in this study suffer from burnout, as have other Italian studies [26,30]. In particular, 30% of PPOs showed high levels of emotional exhaustion, 60% showed high levels of depersonalisation. In particular, almost all participants obtained high scores in the dimension of personal realization. Typically, this dimension is inversely related to burnout, but in this study, only 17% presented lower levels of personal realisation. We found that personal realisation is positively correlated with human traits attribution to colleagues: this could mean that a positive and human relationship with colleagues promotes personal realisation at work. Another hypothesis is that personal realisation could be a form of resiliency in work context. However, this is an interest result and it could be useful to go deeper in future studies. Looking at the results, it can be said that 25 participants, about 30% of the sample, showed burnout, with high levels of both emotional exhaustion and depersonalisation. Child-related concern, survival and coping beliefs and responsibility to family were the most important reasons for living, whereas survival and coping beliefs seemed to be protective factors against exhaustion and depersonalisation. It is important to underscore that these two last dimensions are positively related to fear of suicide. This result suggested that it could be present in PPOs suffering from these burnout variables, a possible suicide ideation expressed in form of fear of suicide. 

Furthermore, the hypothesis about dehumanisation was confirmed, because, indeed, PPOs attribute non-human traits (‘impetus’, ‘impulsiveness’, ‘instinct’ and ‘impulses’ versus ‘reasoning’, ‘thinking’, ‘cognitive abilities’ and ‘moral sense’) more to prisoners than to colleagues. However, a significant negative correlation between dehumanisation and burnout is not found. All this means that in this group, dehumanisation is not a psychological instrument that is useful for increasing motivation for work and for the quality of one’s performance, as Capozza et al. [17] found with respect to health professionals. The results show that Child-Related Concern is one of the most valorised protective factors, together with Responsibility to Family and Survival and Coping Beliefs, indicating the importance of close relationships and positive self-esteem as reasons for wanting to live. It can be also noticed that Survival and Coping Beliefs are negative related to Emotional Exhaustion and Depersonalisation. This means that as work tension and discomfort increase, coping and management abilities in everyday life decrease. The result about the effect of the Survival and Coping Beliefs subscale on burnout, i.e., that it is a protective factor against burnout, as confirmed by other studies. In particular, a high score on the emotional exhaustion scale is associated with anxiety, introversion and intolerance [41], and the SCB subscale is predictive of suicidal risk [42]. Furthermore, Linehan et al. [36] discovered a correlation between recent suicidal ideations and low scores in SCB, whereas Rietdijk et al. [43] confirmed that, on the one hand, the SCB is predictive of parasuicidal and self-injury behaviours, while on the other hand, it is correlated to depression and introversion.

With respect to the personal variables, it is important to underscore that, in the long run, work turned out to be a reason for PPOs’ exhaustion. In fact, those who have been working longer are more exhausted and tense than those who have been working for less time. Indeed, organisations and institutions are increasingly recognising the need to consider the well-being of their workers seriously. The more progressive enterprises promote the valorisation of their workers because they understand that people are their most important resource, the better they work. In other situations, this attitude is just the beginning, and it runs in parallel with the consciousness that many workplace problems derive from a lack of commitment to the needs of their workers.

The first limitation of this study was the participation of PPOs, which did not reach the expected number (150). Another limitation was the almost total absence of a female sample due to the length of waiting time to obtain the necessary authorization to enter the female prison: the imbalance in gender distribution among participants is a factor that may affect the variables taken into account, future research will have to balance the number of men and women. 

Regarding possible developments in this area of research, additional descriptions of the dehumanisation of the inmates could be useful. It could be useful to explore the relation between personal realisation and resiliency. Further studies also need to gather information about the work conditions of all the penitentiary system that is intended as the organisational community (including not only the PPOs, but also the medical staff, educators and all who work in prisons).

## 5. Conclusions

The need to prevent and consider burnout troubles is quite important for penitentiary officers. It is necessary to adopt programmes that target specific health issues in the workplace, in particular those related to suicide risk (considered as a lack of reasons for living) and depersonalisation.

Indeed, the recent and growing transformation of the role of the penitentiary police shows itself through the modification of the relationship between the prisoner and the officer: this relationship, in fact, is no longer something exceptional, an isolated case, but is part of everyday working life. Working in direct contact with prisoners, as can be seen in this study, might not be perceived as an opportunity or a strategy to pursue the professional goal of re-education; indeed, it could be a cause for concern. The presence of burnout among polices and their dehumanising attitude towards prisoners evidently indicates their noteworthy psychological distress, from which the reinforcement of the in-group relationships derives. 

Furthermore, these professionals are caught up in a major contradiction, because on the one hand, they must ensure the containment and segregation of prisoners, but on the other hand, they have to ensure their re-education as well. To reduce their work-related stress, dehumanisation and depersonalisation and to improve their intrinsic motivation and well-being, it is necessary to implement training activities that offer useful psychological and qualified instruments to cope with the difficulties that the relationship with prisoners causes. To develop individual communication skills and abilities to manage critical events, it may be useful to involve the intervention of psychologists who can create spaces in which PPOs can share experiences, goals and strategies and to proceed with regular supervision of the activities. These kinds of training are also useful to increase protective factors to suicide risk, that resulted to be significantly related to emotional exhaustion at work. In fact, if we act in ways that encourage PPOs to manage stress situations at work, we can promote coping strategies and well-being in family life, as long as they become important protective factors.

A lack of recognition of the importance of workers’ well-being may give rise to workplace difficulties such as health and mental disorders. Thus, this study showed the necessity to work in this field, improving psychological interventions that can improve relational competencies and the climate of work. Furthermore, in Italy, all this seems to be unavoidable because of the presence of many prisoners with mental health-related problems.

## Figures and Tables

**Table 1:** Participants Characteristics.

Personal Variables	N	%	Mean (SD)	Work Variables	N	%	Mean (SD)
Gender				Length of service (years)			
Male	73	85		1–34			16.5 (9.6)
Female	5	6		Presence of work shifts			
Missing value	8	9		No	28	33	
Age (years)				Yes	58	67	
25–55	85	99	40.3 (8.6)	Working time (hours/week)			
Missing value	1	1		30–70			40.1 (5.3)
Education				Working time with prisoners (%)			
Low	36	42		1–100			61.9 (35.0)
Middle-High	50	58					
Geographic area of origin							
North Italy	18	21					
South-central Italy	63	73					
Missing value	5	6					
Married/Cohabitant							
No	24	28					
Yes	62	72					
Children							
No	30	35					
Yes	56	65					
Religion							
None	10	12					
Christian	76	88					
Religious practice							
No	63	73					
Yes	23	27					

**Table 2:** Descriptive statistics (range, mean, standard deviation, Cronbach’s alpha) and correlations between all study variables (N = 86).

Study Variables	Mean (SD)	1	2	3	4	5	6	7	8	9	10	11	12	13	14
1.Emotional Exhaustion	16.57 (13.00)	-													
2.Depersonalization	7.53 (6.78)	0.63 ***	-												
3.Personal Realization	30.67 (9.18)	−0.22 *	−0.12	-											
4.Survival and Coping Beliefs	4.90 (0.75)	−0.39 ***	−0.21~	0.14	-										
5.Responsibility to Family	4.28 (1.04)	0.12	0.23 *	0.12	0.51 ***	-									
6.Child-Related Concern	5.05 (1.06)	−0.01	0.07	0.05	0.45 ***	0.66 ***	-								
7.Fear of Suicide	2.43 (1.13)	0.19 ~	0.36 **	−0.20 ~	0.10	0.41 ***	0.13	-							
8.Fear of Social Disapproval	2.53 (1.53)	0.03	0.26 *	−0.13	0.28 **	0.45 ***	0.26 *	0.69 ***	-						
9.Moral Objection	3.14 (1.25)	−0.04	0.10	0.15	0.48 ***	0.57 ***	0.39 ***	0.48 ***	0.51 ***	-					
10.TDRS Total score	3.11 (1.01)	0.10	−0.01	−0.14	−0.13	−0.10	−0.25 *	0.01	−0.07	−0.23 *	-				
11.Ingroup Attribution of HT	3.88 (0.86)	−0.16	0.00	0.24 *	0.02	−0.02	0.08	−0.22 *	−0.13	0.00	−0.11	-			
12.Outgroup attribution of HT	2.63 (0.82)	−0.01	0.10	0.02	0.05	0.09	0.11	0.08	0.13	0.14	−0.14	−0.12	-		
13.Ingroup attribution of N-HT	2.84 (0.86)	0.13	0.15	0.01	−0.03	−0.06	0.00	−0.01	−0.02	−0.05	−0.01	−0.06	0.34 **	-	
14.Outgroup attribution of N-HT	3.62 (0.74)	0.12	0.09	0.08	0.11	0.19	0.22 *	0.04	0.05	0.07	0.01	0.25 *	−0.10	0.33 **	-
Study variables		1	2	3	4	5	6	7	8	9	10	11	12	13	14

Note. HT = Human Traits; N-HT = Non-Human Traits. ~ *p* < 0.10; * *p* < 0.05; ** *p*< 0.01; *** *p* < 0.001.

**Table 3:** Regression analyses results to evaluate the impact of personal variables, work variables and RFL variables on MBI subscales.

	Emotional Exhaustion		Depersonalisation		Personal Realisation	
Variable	Beta	R-Square	Beta	R-Square	Beta	R-Square
Model 1: Personal variables		0.05		0.06		0.06
Age (years)	0.15		0.06		0.03	
Married/Cohabitant (0 = No; 1 = Yes)	0.12		0.17		−0.19 ~	
Religious practice (0 = No; 1 = Yes)	−0.09		−0.19 ~		0.18	
Model 2: Personal and Work variables		0.10		0.17 **		0.08
Age (years)						
Married/Cohabitant (0 = No; 1 = Yes)	0.08		0.13		−0.17	
Religious practice (0 = No; 1 = Yes)	−0.13		−0.25 *		0.16	
Prison structure (0 = CC; 1 = CR)	−0.09		−0.17		0.02	
Length of service (years)	0.26 *		0.21 ~		0.01	
Working time (hours/week)	0.07		0.22 *		0.16	
Model 3: Personal, Work and RFL variables		0.36 ***		0.37 ***		0.14
Age (years)						
Married/Cohabitant (0 = No; 1 = Yes)	−0.01		0.10		−0.19	
Religious practice (0 = No; 1 = Yes)	−0.11		−0.20 *		0.12	
Prison structure (0 = CC; 1 = CR)	−0.12		−0.17 ~		0.03	
Length of service (years)	0.19~		0.11		0.05	
Working time (hours/week)	0.06		0.17 ~		0.13	
Survival and coping beliefs	−0.56 ***		−0.39 **		0.07	
Responsibility to family	0.34 **		0.22 ~		0.17	
Fear of suicide	0.16		0.24 ~		−0.12	
Fear of social disapproval	−0.11		0.05		−0.15	

Note. The variable Age was dropped in model 2 and in model 3 because it overlapped with the new variable, length of service, included in the model 2 as a work variable (correlation between the two variables was 0.94). ~ *p* < 0.10; * *p* < 0.05; ** *p* < 0.01; *** *p* < 0.001.

## Supplementary Materials

The following are available online at https://www.mdpi.com/1660-4601/17/9/3117/s1, Table S1: Descriptive statistics and correlations for all study variables (male participants N = 73), Table S2: Regression analyses results to evaluate the impact of personal variables, work variables and RFL variables on MBI subscales (male participants N = 73).
