# Supplementary material for: Burnout, Reasons for Living and Dehumanisation among Italian Penitentiary Police Officers
Source: Int J Environ Res Public Health. 2020 Apr 30;17(9):3117. doi: 10.3390/ijerph17093117 (PMC7246835; doi:10.3390/ijerph17093117)
Supplement: Supplementary file 1 [file ijerph-17-03117-s001.pdf]

Table S1 - Descriptive statistics and correlations for all study variables (male participants N=73)

Study variables	Mean (SD)	1	2	3	4	5	6	7	8	9	10	11	12	13	14
1.Emotional Exhaustion	15.33 (12.14)	-													
2.Depersonalization	7.23 (6.72)	0.64***	-												
3.Personal Realization	31.26 (8.78)	-0.22~	-0.14	-											
4.Survival and Coping Beliefs	4.97 (0.66)	-0.22~	-0.19	0.06	-										
5.Responsibility to Family	4.32 (1.04)	0.19	0.23~	0.09	0.53***	-									
6.Child-Related Concern	5.17 (0.98)	0.01	0.06	0.07	0.51***	0.64***	-								
7.Fear of Suicide	2.32 (1.06)	0.12	0.30*	-0.19~	0.27*	0.43***	0.16	-							
8.Fear of Social Disapproval	2.51 (1.55)	-0.01	0.18	-0.11	0.38**	0.44***	0.26*	0.68***	-						
9.Moral Objection	3.12 1	-0.04	0.01	0.13	0.54***	0.54***	0.40***	0.44***	0.49***	-					
10.TDRS Total score	3.05 (1.00)	0.04	0.03	-0.09	-0.02	0.01	-0.16	0.07	-0.02	-0.10	-				
11.Ingroup Attribution of HT	3.92 (0.89)	-0.18	0.01	0.24*	-0.02	-0.04	0.09	-0.22~	-0.11	0.04	-0.14	-			
12.Outgroup attribution of HT	2.65 (0.87)	0.04	0.11	0.00	0.02	0.06	0.10	0.07	0.10	0.10	-0.09	-0.09	-		
12.Ingroup attribution of N-HT	2.82 (0.89)	0.13	0.11	0.05	-0.04	-0.13	-0.07	-0.08	-0.07	-0.09	0.00	-0.03	0.35**	-	
14.Outgroup attribution of N-HT	3.61 (0.77)	0.12	0.12	0.10	0.13	0.20~	0.24*	0.08	0.11	0.13	-0.04	0.23~	-0.06	0.34**	-
Study variables		1	2	3	4	5	6	7	8	9	10	11	12	13	14

Note. HT=Human Traits; N-HT=Non-Human Traits
~p<0.10; *p<0.05; **p<0.01; ***p<0.001

Table S2 - Regression analyses results to evaluate the impact of personal variables, work variables and RFL variables on MBI subscales (male participants N=73)

	Emotional Exhaustion		Depersonalization		Personal Realization	
Variable	Beta	R-square	Beta	R-square	Beta	R-square
Model1: Personal Variables		0.03		0.05		0.04
Age (years)	0.15		0.10		0.00	
Married/Cohabitant (0=No; 1=Yes)	0.05		0.14		-0.13	
Religious practice (0=No; 1=Yes)	-0.04		-0.17		0.17	
Model2: Personal Variables and Work Variables		0.13~		0.20**		0.07
Age (years)[1]						
Married/Cohabitant (0=No; 1=Yes)	0.04		0.14		-0.11	
Religious practice (0=No; 1=Yes)	-0.09		-0.25*		0.15	
Prison structure (0=CC; 1=CR)	-0.17		-0.21~		0.01	
Length of service (years)	0.27*		0.23~		-0.02	
Working time (hours/week)	0.17		0.26*		0.16	
Model3: Personal Variables, Work Variables and RFL variables		0.26*		0.34**		0.13
Age (years)[1]						

Married/Cohabitant (0=No; 1=Yes)	-0.02	0.11	-0.13
Religious practice (0=No; 1=Yes)	-0.09	-0.21~	0.11
Prison structure (0=CC; 1=CR)	-0.16	-0.19~	0.00
Length of service (years)	0.22~	0.13	0.03
Working time (hours/week)	0.11	0.18	0.16
Survival and Coping Beliefs	-0.37**	-0.40**	0.06
Responsibility to Family	0.32*	0.24~	0.13
Fear of Suicide	0.12	0.21	-0.22
Fear of Social Disapproval	-0.17	0.02	-0.09

Note. [1]This variable was dropped because overlapped with the new variable, Lenght of service, included in the model as work variable (correlation between the two variables was 0.95)

~p<0.10; *p<0.05; **p<0.01; ***p<0.001
